# Identification and Characterization of Phytocyanin Family Genes in Cotton Genomes

**DOI:** 10.3390/genes14030611

**Published:** 2023-02-28

**Authors:** Muhammad Bilal Tufail, Muhammad Yasir, Dongyun Zuo, Hailiang Cheng, Mushtaque Ali, Abdul Hafeez, Mahtab Soomro, Guoli Song

**Affiliations:** 1 State Key Laboratory of Cotton Biology, Institute of Cotton Research of the Chinese Academy of Agricultural Sciences, Anyang 455000, China; 2 The Key Laboratory for Quality Improvement of Agricultural Products of Zhejiang Province, College of Advanced Agricultural Sciences, Zhejiang A&F University, Hangzhou 311300, China; 3 Department of Agronomy, Sindh Agriculture University Campus Umerkot, Sindh 69100, Pakistan

**Keywords:** phytocyanins (PCs), cotton, fibre, gene expression profile

## Abstract

Phytocyanins (PCs) are a class of plant-specific blue copper proteins that have been demonstrated to play a role in electron transport and plant development. Through analysis of the copper ligand residues, spectroscopic properties, and domain architecture of the protein, PCs have been grouped into four subfamilies: uclacyanins (UCs), stellacyanins (SCs), plantacyanins (PLCs), and early nodulin-like proteins (ENODLs). The present study aimed to identify and characterise the PCs present in three distinct cotton species (*Gossypium hirsutum*, *Gossyium arboreum*, and *Gossypium raimondii*) through the identification of 98, 63, and 69 genes respectively. We grouped PCs into four clades by using bioinformatics analysis and sequence alignment, which exhibit variations in gene structure and motif distribution. PCs are distributed across all chromosomes in each of the three species, with varying numbers of exons per gene and multiple conserved motifs, and with a minimum of 1 and maximum of 11 exons found on one gene. Transcriptomic data and qRT-PCR analysis revealed that two highly differentiated PC genes were expressed at the fibre initiation stage, while three highly differentiated PCs were expressed at the fibre elongation stage. These findings serve as a foundation for further investigations aimed at understanding the contribution of this gene family in cotton fibre production.

## 1. Introduction

Phytocyanins (PCs) are ancient blue copper-binding proteins (BCPs) in plants that serve as electron transporters and bind to single type I copper atoms [1,2,3]. All PCs possess a structural motif characterised by an eight-stranded Greek key barrel or sandwich fold, which includes two conserved cysteine residues that are linked by disulphide bridges. Additionally, some members of this protein class possess four copper ligands, composed of two histidine residues, one cysteine residue, and either a methionine or glutamine residue [4,5]. A bioinformatics analysis revealed that the plant species *Arabidopsis thaliana*, *Oryza sativa*, and *Brassica rapa* possess 38, 62, and 84 PC genes, respectively [6,7,8]. The four subfamilies of the PCs are known as uclacyanins (UCs), stellacyanins (SCs), plantacyanins (PLCs), and early nodulin-like proteins (ENODLs), and they are separated based on traits such as copper ligand residues, spectroscopic and redox properties, and the domain organisation of the protein [9,10]. A plastocyanin-like domain is found in all PC genes (PCLD). The copper ligand residues in the domains of UCs and PCs are the same (two His, one Cys, and one Met). In contrast to PLCs, UCs are chimeric glycoproteins, whereas PLCs are not [9]. Similar to this, SCs have a glycoprotein-like domain with two His, one Cys, and one Gln in their copper-binding domain [11,12]. N-linked glycosylation sites are present in SCs and UCs via the asparagine (Asn) residue, as along with O-linked glycosylation sites via the serine (Ser) and hydroxyproline (Hyp) residues. ENODLs possess structural features that are similar to those of the other three subfamilies. However, an analysis of ENODLs from the plant species *Arabidopsis* and rice revealed that the majority of these proteins are chimeric arabinogalactan proteins (AGPs). Additionally, ENODLs lack the specific amino acid residues required for copper binding [6,7,10,13].

ENODLs are transcribed during the early stages of root nodule development in leguminous plants. Given this temporal expression pattern, it is proposed that they play a role in the process of cell differentiation and modification of the cell wall during nodulation. [13,14,15]. Nodulins is the original name for plant genes whose expression is activated in legumes by *Rhizobium* bacteria upon nodulation. Several of these are essential to the development of symbiosis. ENODLs can also be identified in non-leguminous plants such as *Arabidopsis*, maize, rice, and poplar [16]. It is noteworthy that homologues of nodulin genes have been identified in the genomes of certain plant species that lack the ability to form root nodules. These findings suggest that nodulin-like genes may have played a role in the evolution of plants’ physiology. The majority of the nodulin-like genes that have been studied thus far are involved in the transport of various solutes throughout plant development. Additionally, the involvement of several ENODLs in interactions between plants and pathogenic microorganisms emphasises the importance of solute transport in plants’ innate immunity [16].

Cotton is a highly valuable crop, primarily because of its mature fibres that are enriched with cellulose. These fibres are derived from the epidermal cells of the ovules, and they are single-celled trichomes. The ability of cotton fibres to import and metabolise sucrose is considered to be a key determinant of sink strength in tissues such as growing cotton seeds and fibres [17]. The trichomes (i.e., leaf hairs, root hairs, and cotton fibres) of *Arabidopsis*—a crop plant that is distinct from other crop plants—are characterised by their elongated single-cell morphology, in contrast to the majority of plant trichomes, which are multicellular. The mechanism underlying the presence of elongated unicellular trichomes in multiple species is currently unknown, and it remains to be determined whether it is a result of a shared regulatory mechanism or a common adaptive response to similar ecological conditions [18]. The genus *Gossypium*, belonging to the family Malvaceae, is composed of 50 recognised species, of which 45 are diploids. These diploids are further divided into three geographical groups and corresponding subgenera, namely, *Sturtia*, *Houzingenia*, and *Gossypium*. Additionally, there are five tetraploid species included in the subgenus *Karpas* [19] Both lint and fuzz are types of cotton fibres produced by the widely cultivated allopolyploid *Gossypium hirsutum* L. (AD genome), as well as two potential A genome progenitors—*Gossypium herbaceum* L. and *Gossypium arboreum*—during distinct waves of epidermal differentiation. Lint initiation typically begins prior to or on the day of anthesis, and a secondary wave of fuzz fibres emerges a few days later from rapidly growing spherical cells [20]. Fuzz and lint fibres are traditionally distinguished by the fact that the former are 90% shorter and more challenging to separate from the cottonseed. The residual fibre on the cottonseed after the removal of lint fibres is referred to as fuzz. Until recently, besides a few traditional genetic investigations [21], little has been known about the molecular control of fuzz initiation.

Each long cotton “lint” fibre originates from a single epidermal cell located on the surface of the ovule, which undergoes significant polar expansion and thickening of the cell wall to form the elongated and reinforced dead fibre. Fibre morphogenesis involves a series of steps, including initiation, elongation, primary wall remodelling, secondary wall synthesis, and maturation. These differentiation processes, which typically take at least 50 days, directly determine the properties of the cotton fibre [22]. The development of certain ovule epidermal cells into fibre initials, or spherical protrusions on the ovule surface, represents the initial stage of cotton fibre morphogenesis. In modern cultivars, fibre initiation typically begins on the day of anthesis and continues for at least 5 days following anthesis (DPA), by which time each ovule contains over 16,000 fibre initials (including immature elongating fibres) [23,24]. The central vacuole develops as the initials grow, and the nucleus moves from the base to the centre of the growing fibre. Five *G. hirsutum* cultivars showed a positive correlation between the density of fibre initials and the lint % and lint index at 0 and 1 DPA [25]. During cotton domestication, humans selected cultivars that exhibited higher fibre density and more synchronous fibre initiation through a process of selection [26]. Generation of turgor pressure within the central vacuole drives the expansion of fiber initials [27].

The PC gene family is known to play multiple roles in plants, including the transport of electrons. In the present study, we sought to investigate its function in fibre development in cotton. To produce the highest-quality fabric, the finest cotton fibre is required. There is currently no genome-wide study of the PC gene family in cotton. However, this gene family has been previously studied in other crop species, such as *O. sativa*, *A. thaliana*, and *B. rapa*. We conducted a bioinformatics analysis to study the gene structures, biological functions, cis-regulatory elements, conserved motifs, chromosomal positions of genes, and expression patterns. The main objective of this study was to facilitate the research related to cotton fibre development.

## 2. Materials and Methods

### 2.1. Genome-Wide Identification, Sequence Alignment, and Phylogenetic Tree

The data of *G. hirsutum*, *G. arboreum*, *G. raimondii*, and *A. thaliana* were obtained from the Cotton Functional Genomic Database (http://www.cottonfgd.org (accessed on 28 November 2019)) and the National Center for Biotechnology Information (https://www.ncbi.nlm.nih.gov/ (accessed on 28 November 2019)), and multiple protein sequence alignment was performed using ClustalW [28] for *G. hirsutum*, *G. arboreum*, *G. raimondii*, and *A. thaliana*. A phylogenetic tree was constructed using the *p*-distance method in MEGA7 [29], with 1000 bootstrap replications. Additional characteristics of PC genes, including protein length (aa) and molecular weight (kDa), were analysed using the Cotton Functional Genomic Database (http://www.cottonfgd.org/ (accessed on 28 November 2019)) [30,31]. By utilising the Cotton Functional Genomic Database (CottonFGD) (https://cottonfgd.org/ (accessed on 28 November 2019)), we obtained biochemical information about the PC proteins in *G. hirsutum* L, including their isoelectric points (pIs), molecular weights (MWs), grand average of hydropathy, and charge [30,31].

### 2.2. Chromosomal Mapping and Collinearity Analysis

The genomic locations of all PC genes were obtained from the gff3 files of three cotton species available on the Cotton Functional Genomic Database (http://www.cottonfgd.org/ (accessed on 19 October 2019)). The TBtools software was utilised to determine the chromosomal locations of these genes. The circle gene viewer model of the TBtools software was employed to visualise the collinearity between homologous gene pairs [32].

### 2.3. Gene Structure, Conserved Motifs, and Cis-Regulatory Elements

The full-length protein sequences of *Arabidopsis* and *G. hirsutum* were aligned using ClustalW, and an NJ tree was constructed using MEGA 7.0 [29] with the aforementioned procedure and settings. A Perl script was employed to extract exon positions from the gff3 file, and the TBtools software was used to display the gene structure and identify conserved patterns (http://meme-suite.org/ (accessed on 27 October 2019)). The 5′ untranslated regions, 2000 bp upstream of the transcription start site (TSS) in the genomic DNA sequence of PCs as promoters, were extracted from the Cotton Functional Genomic Database (CottonFGD) (https://cottonfgd.org/ (accessed on 25 October 2019)). These genomic DNA sequences were then submitted to the PlantCARE database (https://bioinformatics.psb.ugent.be/webtools/plantcare/html (accessed on 25 October 2019)) to predict cis-acting elements in the promoter regions of the PC genes [33].

### 2.4. Selection Pressure

To evaluate the Ka/Ks ratio and infer the selection pressure between the genes of each pair in the genomes and subgenomes, the coding sequences (CDSs) of homologous gene pairs of *G. hirsutum* (NAU), *G. arboreum* (CRI), and *G. raimondii* (JGI) were analysed using the TBtools software [32].

### 2.5. Transcriptomic Profiling of the Phytocyanin Gene Family in Cotton

The raw RNA-Seq data were taken from the published paper [34]. We selected the PCs with RPKM ≥ 5 (reads per kilobase million) for further expression analysis. We collected the fibre of *G. hirsutum* L. TM-1 at different DPA (days post-anthesis) levels—3 DPA, 10 DPA, 15 DPA, and 20 DPA—with three biological replicates for each stage. All samples were immediately frozen in liquid nitrogen and stored at −80 °C. The RNAprep Pure Plant Kit (TIANGEN, Beijing, China) was used to separate total RNA, which was then subjected to DNase I treatment to remove genomic DNA. The isolated RNA was reverse-transcribed using the PrimeScript^®^ RT Reagent Kit (Perfect Real Time, Takara Biotechnology Co., Ltd., Dalian, China) to produce first-strand cDNA for transcriptome analysis. The fragments per kilobase of exon model per million mapped reads (FPKM) technique was used to standardise the data. Utilising the ABI 7500 rapid Real-Time PCR instrument, qRT-PCR was carried out (Applied Biosystems, USA) [35]. The primers used for qRT-PCR are shown in Table S1 Primer pairs of four PC genes were designed using Oligo 7 [36], and the 2^−ΔΔCt^ method was used to calculate the gene expressions [37,38].

## 3. Results

### 3.1. Genome-Wide Identification, Sequence Alignment, and Phylogenetic Tree

**Figure 1:**
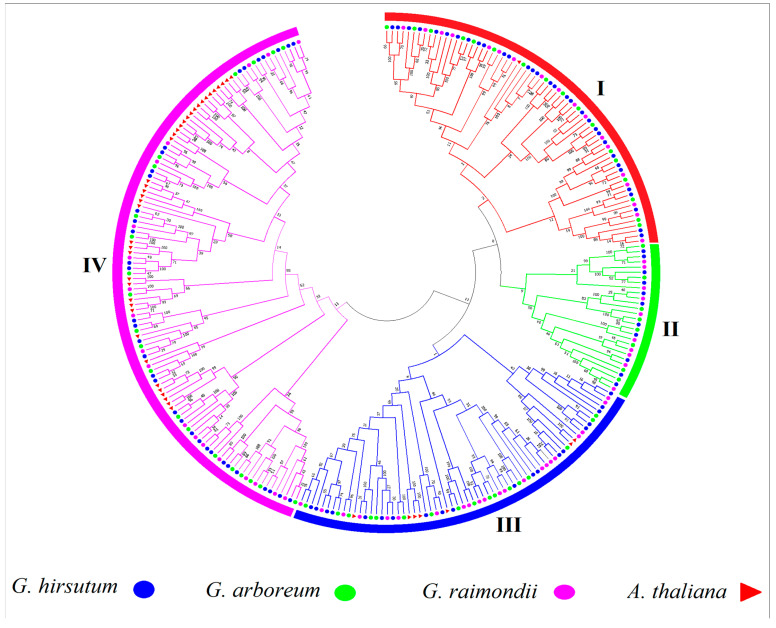
Phylogenetic tree of 277 PC genes from *G. hirsutum* (A—subgenome 48, D—subgenome 50), *G. arboreum* (63), *G. raimondii* (69), and *Arabidopsis* (47); 100 replicates show 1000 bootstraps as a percentage. Different line colours indicate different clades of PCs. *G. hirsutum* genes are marked with blue circles, *G. arboreum* with green, *G. raimondii* with purple and *A. thaliana* with red triangles.

Protein sequences of *G. hirsutum*, *G. raimondii*, and *G. arboreum* were downloaded from CottonFGD (http://www.cottonfgd.org/ (accessed on 29 September 2019)). Using the hidden Markov model (HMM) profile of the PPIase domain PF02298, a total of 98 *G. hirsutum*, 63 *G. arboreum*, and 69 *G. raimondii* genes were identified. The characteristics of the PCs of *G. hirsutum* were then identified, including genomic length (bp), protein length (aa), CDS length (bp), locus ID with corresponding chromosome number, strand polarity, start and end points, predicted isoelectric points (PI), predicted masses, and protein molecular weights (MW), as shown in Tables S2 and S3. Retrieving the information of PC genes in *G. hirsutum* revealed that *Ga06G1182*, which was detected on chromosome A06 in *G. arboreum*, coded the smallest protein of 60 amino acids (aa), with a molecular weight of 6.566 kDa. Meanwhile, *Gh_A03G1381*, which was identified on chromosome *At*03, coded the largest protein among all PC members in three *Gossypium* species, having 404 aa, with a molecular weight of 45.973 kDa. The isoelectric point (PI) of the PC genes varied from 3.835 (*Gh_A13G2127*) to 10.679 (*Gh_A01G1490*). To analyse the evolutionary relationships of the PC genes, we constructed a neighbour-joining (NJ) phylogenetic tree by the multiple alignment of 230 phytocyanins of *G. hirsutum* (98), *G. arboreum* (63), and *G. raimondii* (69) with 47 genes of *A. thaliana*. The phylogenetic tree was divided into four clades containing three cotton species and *A. thaliana* genes. All genes were positioned into different clades according to their majority and similarity in each clade. As shown in the phylogenetic tree (Figure 1), genes of *A. thaliana* (marked as red triangles) were mostly found in Clades III and IV. Early nodulin-like proteins were present in Clades I and IV, along with only one gene shown in Clade II. The majority of the *A. thaliana* genes were found with early nodulin-like proteins in the same clades (i.e., Clades III and IV). This showed a great evolutionary relationship among these genes. Uclacyanins (UCs) and stellacyanins (SCs) were scattered in different clades.

### 3.2. Chromosomal Mapping and Collinearity Analysis

**Figure 2:**
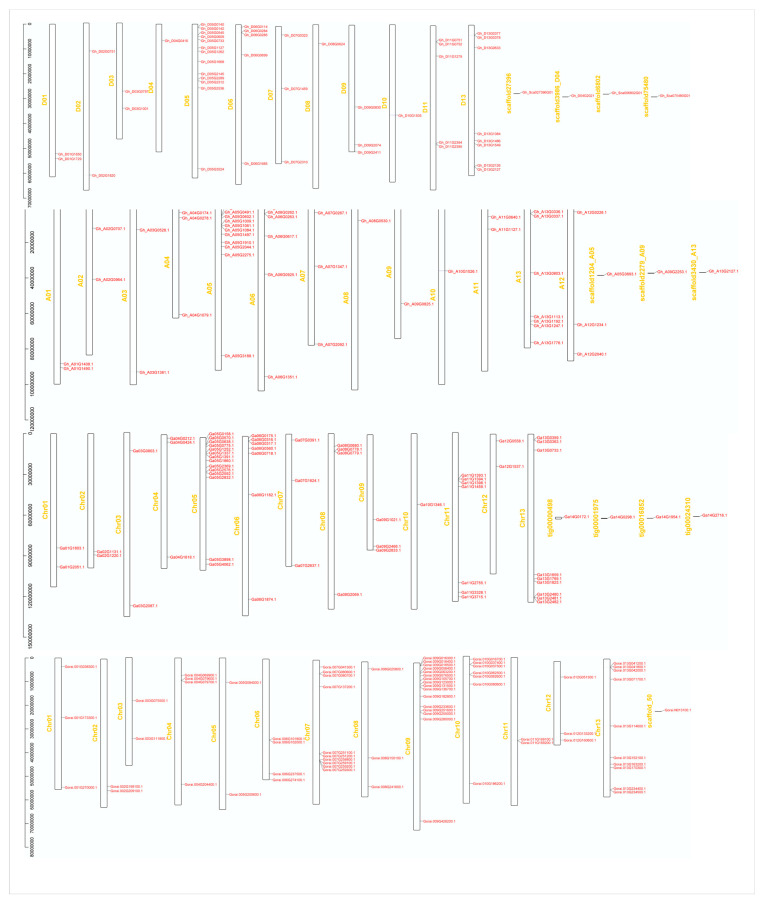
Chromosomal distribution of PCs on *G. hirsutum*, *G. arboreum*, and *G. raimondii*. The chromosomal IDs can be seen on the right-hand side of the vertical bars, while chromosome numbers are on the left. Scaffolds are shown at the end of each row.

**Figure 3:**
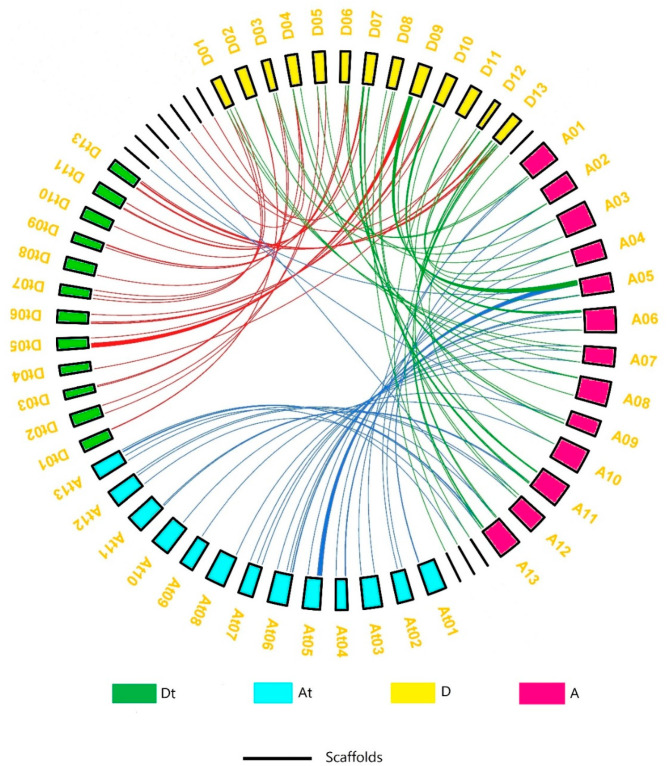
Collinearity analysis of PC genes between the *AtDt* (*G. hirsutum*), A (*G. arboreum*), and D (*G. raimondii*) genomes. Blue lines show homology between At (*G. hirsutum*) and A (*G. arboreum*); red lines show homology between Dt (*G. hirsutum*) and D (*G. raimondii*); green lines show homology between A (*G. arboreum*) and D (*G. raimondii*).

To determine the division of PC genes on different chromosomes of cotton, we constructed the chromosomal map with information taken from three cotton genomes (Figure 2). The results showed that most of the 230 genes were scattered irregularly on 13 chromosomes, while 12 genes (*Gh_D04G2021*, *Gh_Sca006802G01*, *Gh_Sca027396G01*, *Gh_Sca075480G01*, *Gh_A05G3693*, *Gh_A09G2253*, *Gh_A13G2127*, *Ga14G0172*, *Ga14G0298*, *Ga14G1954, Ga14G2716*, and *Gorai.N013100*) were located on different scaffolds. In *G. raimondii*, chromosome 09 was found to have the highest number of PC members, with 16 genes, followed by chromosomes 07 and 13, with 10 genes each; the lowest number of genes (2) was found on chromosomes 03, 05, and 11. In *G. arboreum*, chromosome 05 had the highest number of genes (14), followed by 9 genes on chromosome 13; the lowest number of genes (1) was found on chromosome 10. In *G. hirsutum* (AtDt genome), the highest number of genes were found on chromosome 05, with 12 in the At subgenome and 13 in the Dt sub-genome. Only one PC member was found on chromosomes At08, At09, At10, Dt04, Dt08, and Dt10. The collinearity of the PC genes on the chromosomes of the *Gossypium* species was examined, and the results are shown in Figure 3. The results showed that the chromosomes on which the PC genes were mapped had pairwise collinearity of PC genes. A number of genes present in the At and Dt scaffolds were found to be aligned with their homologues in the A and D genomes, indicating that the scaffolds and chromosomes where these PCs are located are homologous. Genes from *G. arboreum* and *G. raimondii* were found to overlap with the At and Dt subgenomes of *G. hirsutum*, respectively.

### 3.3. Gene Structure, Conserved Motifs, and Cis-Regulatory Elements

To determine the gene structures of PCs, the intron–exon structures of 230 cotton genes were analysed using the TBtools software, as shown in Figure S1. The analysis revealed that seven genes *(Gh_A05G3693, Gh_A09G0825, Gh_D05G0140, Gh_D05G2312, Gh_D09G0830, Gh_D11G0751, and Gh_Sca027396G01)* had only one exon, while the gene Gh_A03G1381 had the largest number of exons, with 11. Twenty motifs were identified using (http://meme-suite.org/ (accessed on 5 October 2019)). Motifs 1 and 2 were present in all genes, except for 10 and 8 genes, respectively (Figure S2). The promoter regions of the PC gene family were found to contain significant numbers of cis-regulatory elements. The enrichment of MYB, TATA-box, and CAAT-box cis-regulatory elements was discovered through analysis of cis-regulatory elements (Figure S3). Other enriched cis elements were also found, such as MYC, ABRE, Box 4 (part of a conserved DNA module involved in light responsiveness), GATA-motif 32, wun-motif, and W-box. These cis-regulatory components may work together, depending on their individual roles, the settings they are in, and the stages of growth and development.

### 3.4. Selection Pressure

The Ka/Ks ratio is a measure used in genetics to assess the balance between neutral mutations, purifying selection, and positive selection on a set of homologous genes. The ratio is calculated as the number of synonymous substitutions per synonymous site (Ks), divided by the number of non-synonymous substitutions per non-synonymous site (Ka), and it serves as an indicator of selection pressure on a gene [7]. The balance between neutral mutations, purifying selection, and positive selection on a gene can be determined by analysing the Ka/Ks ratio of a set of homologous genes. A Ka/Ks ratio < 1 is indicative of purifying selection pressure, while a ratio of Ka/Ks = 1 represents neutral selection pressure, and a ratio of Ka/Ks > 1 represents positive selection pressure. An analysis of the Ka/Ks ratios of homologous PCs in the three *Gossypium* species revealed that there is purifying selection pressure acting on these species. The Ka/Ks ratio in most of the homologous PCs in *G. raimondii* and *G. arboreum* ranged from 0.06 to 0.9, with the exception of three pairs that showed positive selection pressure. In *G. raimondii* and *G. hirsutum*, the ratio ranged from 0.09 to 0.9, with the exception of four pairs that showed positive selection pressure. In At and Dt of *G. hirsutum*, the ratio ranged from 0 to 0.8, with the exception of five pairs that were under positive selection pressure (Table S4).

### 3.5. Transcriptome Profiling of PCs in Cotton

**Figure 4:**
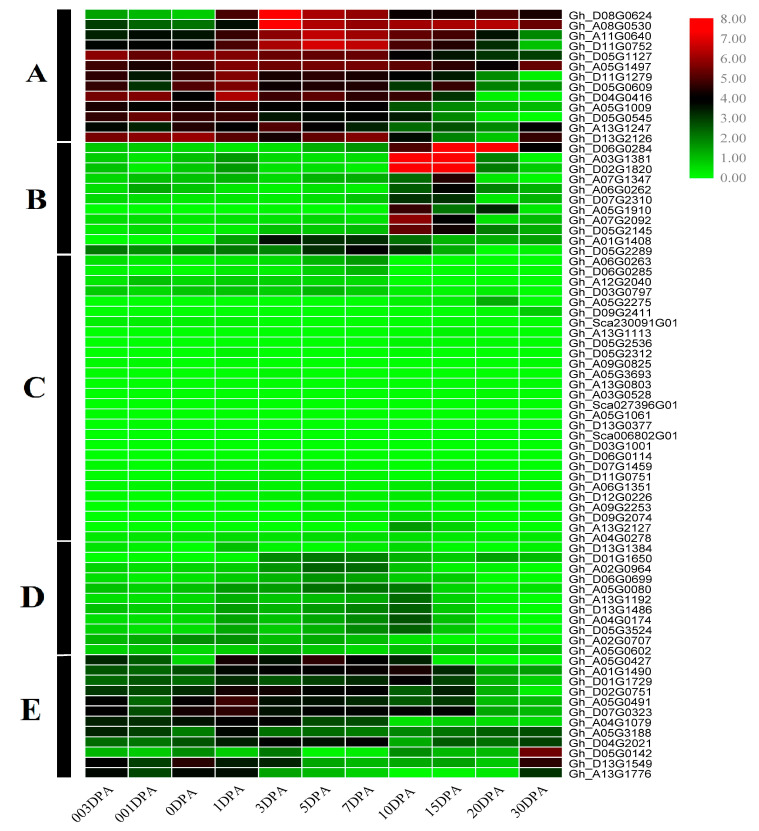
Expression patterns of PC genes during fibre development. A graduated colour scale from green to red is used to denote the transcript levels. The gene expressions are shown at −3, −1, 0, 1, 3, 5, 7, 10, 15, 20, and 30 DPA against their gene IDs on the right-hand side of the heatmap. The results can be classified into five pattern groups A, B, C, D, and E.

Based on RNA-Seq transcriptome data, the expression levels of the PC gene family in *G. hirsutum* were analysed at various stages of fibre development (from −3, −1, 0, 1, 3, 5, 7, 10, 15, 20, and 30 DPA (days post-anthesis)) using RPKM values (Figure 4). The heatmap showed that all genes of the PC family clustered in five patterns, i.e., A, B, C, D, and E. In general, genes within the same subgroup showed similar expression patterns (Figure 4). The group A, which belonged to the subfamily uclacyanin-3, contained highly expressed genes in most of the fibre development stages. However, *Gh_D08G0624* and *Gh_A08G0530*, which were downregulated in the ovule development stages, showed a sharp increase in expression at 3 DPA, suggesting their possible function in fibre initiation as well as in the elongation stage. The members of group B were more distinct than those of group A, downregulated in the fibre initiation and maturation stages, and specifically expressed in the fibre elongation stage (i.e., 10 DPA, 15 DPA, and 20 DPA, respectively). Three genes of group B were found to be highly expressed in 10 DPA, 15 DPA, and 20 DPA fibres; among them, *Gh_A03G1381* and *Gh_D02G1820* were ENODLs, while *Gh_D06G0284* was a copper-binding protein. The genes in groups C and D were found to be downregulated in all fibre development stages. However, the member genes of group E were slightly expressed at different fibre development stages, with the exception of Gh_*D05G0142*—which showed a differential expression pattern at the fibre maturation stage of 30 DPA—and Gh_D13G1549 and Gh_A13G1776, which were specifically expressed in the ovule development and fibre initiation stages (3 DPA, 1 DPA, and 0 DPA) and also at fibre maturation stage at 30 DPA, respectively. The transcriptome profiling (Table S5) of PC genes indicated that some members of this gene family are evolutionarily conserved for their function in different fibre development stages.

### 3.6. qRT-PCR Amplification of Four Homologous PC Gene Pairs

**Figure 5:**
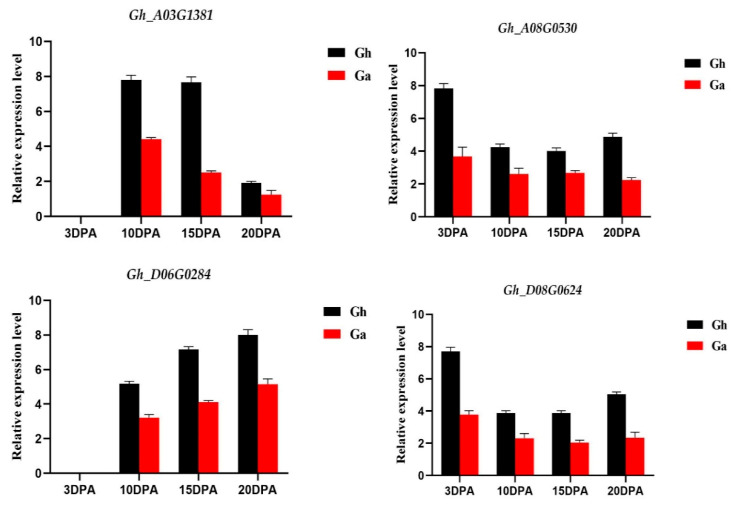
Expression of PCs in different fibre development stages. The expression of genes was determined by qRT-PCR using the total RNA isolated from TM-1 and *G. arboreum*. The error bars indicate the standard error (standard deviation) of three biological replicates.

To verify the differential expression patterns of four selected homologous PC gene pairs from *G. hirsutum* and *G. arboreum*, qRT-PCR was carried out in this study. The results (Figure 5) showed that the overall expression of the PC genes peaked at 20 DPA in Asiatic cotton; however, PC genes showed an exponential expression pattern from 10 to 20 DPA in upland cotton. The average performance of the genes under study in upland cotton was higher than that in the Asiatic cotton fibre development at 10, 15, and 20 DPAs, suggesting that the expression level of upland cotton PC genes mostly increased during the late development of cotton fibres, except for *Gh_D08G0624* and *Gh_A08G0530*, which were highly expressed at 3 DPA. These finding implies that PCs have a probable role in the development of cotton fibres.

## 4. Discussion

A genome-wide search of *G. hirsutum*, *G. arboreum*, and *G. raimondii* resulted in the identification of 230 PCs (98 in *G. hirsutum*, 63 in *G. arboreum*, and 69 in *G. raimondii*). According to a recent study, the two diploid species of *G. raimondii’s* progenitors naturally hybridised to produce the contemporary allotetraploid species (D-genome) [39] and *G. arboreum* (A-genome) [40] 1.7–1.9 million years ago [41]. In comparison to the total number of phytocyanin genes in the *G. hirsutum* A and D genomes, the results of the current investigation showed a loss of quite a significant number of phytocyanins in the *G. hirsutum AtDt* genome. As many as 18 phytocyanin genes in the *At* subgenome and 17 in the *Dt* subgenome of *G. hirsutum* (Figure 3) may have been lost throughout the evolution process after the hybridisation [41]. Gene disruption compared to their orthologous counterparts, premature stop codons, and other factors can cause gene losses [42] and rapid genome reorganization during polyploidization and diploidization processes [43,44,45]. Previous studies have indicated that polyploidization events may result in the loss of homologous genes or changes in gene expression levels, or both [46,47,48,49]. Within four phylogenetic clades, phylogenetic analysis revealed considerable similarity and monophyletic distribution among *Gossypium* species, which may support the conservative evolution style of PC genes.

*G. raimondii* (D-genome) and *G. arboreum* (A-genome) are the closest relatives to *Dt* and *At*, respectively, according to earlier investigations in allotetraploids’ subgenomes. [41]. Every gene in the A or D genomes of *G. hirsutum* has a homologue in the corresponding *At* or *Dt* subgenome [50]. However, we found quite a few PC genes in both the A and D genomes that were not homologous in the related *At* and *Dt* subgenomes. Previous research showed that such homologue loss might be caused by one of two scenarios: either the homologues were lost during the polyploidization process from diploids to tetraploids, or the PC members in each genome began their own distinct evolution after the tetraploids’ creation. The newly developed individuals have no homologues in their relative genomes as a result of this independent evolution process. [41]. Previous research showed that the evolution of the A, D, and *AtDt* genomes did not proceed at the same rate. Allotetraploid cotton evolved more quickly than diploid cotton, according to [41].

The gene structure analysis revealed that seven genes had only a single exon, while a gene identified as *Gh_A03G1381* had 11 exons. It was observed that the gene with the highest number of exons also had the highest expression levels, while genes with only one exon displayed minimal or no expression in the transcriptomic data (Figure S1). 

Plants experience numerous biotic and abiotic stresses during their whole life cycles that negatively affect their growth, productivity, and development [51]. In the presence of these stresses, plants need some potential mechanism that can be switched on in critical circumstances, to support the whole plant life cycle [52]. The output of cotton worldwide is significantly impacted by excessive salt as another important factor [53]. When cis-regulatory elements were identified, it was discovered that the PC genes were particularly rich in crucial cis-regulatory elements that are necessary to protect against harmful environmental pressures. PC genes have been found to contain certain significant regulatory elements, including MYC, MBS, W-box, MYB, ABRE, and G-Box. W-box is necessary to bind WRKY TFs and control gene expression. The role of WRKY TFs in plant defences against chilling, wounding, drought, salinity, and heat stressors is crucial. [54,55,56,57,58,59,60,61]. MYB and MYC have been recognised to play a key role in dehydration responses [62]. ABRE is a crucial regulatory component that improves plants’ ability to withstand salt stress; it is crucial in the reactions to dehydration, salinity stress, and chilling or cold in *Paeonia suffruticosa*, *A. thaliana*, soybeans, and rice [63]. Previous investigations have revealed the G-box in numerous gene promoters, where it participates in plant development, hormone response, and tolerance to fungal infections. These outcomes are consistent with our conclusions. Under particular circumstances and developmental phases, plants may have biological functions—particularly the repeatedly found cis-regulatory elements.

By computing the replacement rates of non-synonymous (Ka) and synonymous sequences, we examined the homologous sequence variation of three cotton species (Ks). The findings revealed that the Ka/Ks ratio of roughly 95% of homologous gene pairs for PC genes in the three cotton species was less than one (Table S3), indicating a strong purifying or stabilising selection in the evolution of PC genes in the genus *Gossypium* and the slow evolution of these genes in these plants. Three pairs of *G. raimondii* and *G. arboreum*, four pairs of *G. raimondii* and *G. hirsutum*, and five pairs in subgenomes of *G. hirsutum* were found to be positively selected (Table S3). It is assumed that the genes with positive selection may have experienced functional differentiation.

The identification of gene function can be facilitated by the analysis of gene expression patterns. In order to examine the PC gene expression profiles during the development of *G. hirsutum* L. fibres, samples of fibres from various developmental stages were collected. In *G. hirsutum* L., the transcriptomic study of the PC genes was conducted (Table S5). The findings demonstrated that, during fibre formation, 17 genes were differentially expressed, while the remaining 58 genes were undifferentiated (Figure 4). To verify the reliability of the transcriptomic data, we selected four genes for qRT-PCR verification; all four genes showed differential expression in different developmental stages. However, these genes showed significant differential expression in *G. hirsutum* as compared to *G. arboreum.* This study provides a comprehensive understanding of the PC gene family in cotton, which will facilitate future research on the roles of these genes in fibre development.

## 5. Conclusions

Ancient blue copper-binding proteins called phytocyanins (PCs) are found in a number of plant species and are crucial for the growth and stress resilience of plants. This was the first study of PC genes in cotton to provide the basic information on respective genes and their function in cotton during fibre development. Cis-regulatory elements showed that PCs also play a role in abiotic stresses. Furthermore, expression profiling analysis based on transcriptomic data and the results of qRT-PCR showed that the PC genes *Gh_D08G0624*, *Gh_A08G0530*, *Gh_A03G1381*, *Gh_D02G1820*, and *Gh_D06G0284* might be potential candidates for cotton fibre development. These investigations will provide valuable insights for the comprehensive characterisation of these genes.

## Supplementary Materials

The following supporting information can be downloaded at: https://www.mdpi.com/article/10.3390/genes14030611/s1, Figure S1: The gene structures of PCs. Gene structure analysis was performed by TBtools software. Figure S2: The conserved protein motifs of the amino acid sequences of PCs were analyzed by the MEME. Figure S3: Cis-regulatory elements. Table S1: A list of primers used in RT qPCR experiments. Table S2: Choromosomes start and end points and transcript length. Table S3: In silico identification and analyses of PC gene family in G. hirsutum. Table S4: The Ka/Ks ratio. Table S5: Transcriptomic data of Phytocyanin genes (PCs).
