# Supplementary material for: Identification and Characterization of Phytocyanin Family Genes in Cotton Genomes
Source: Genes (Basel). 2023 Feb 28;14(3):611. doi: 10.3390/genes14030611 (PMC10048054; doi:10.3390/genes14030611)
Supplement: Supplementary file 1 [file genes-14-00611-s001.zip › Figure S2.pdf]

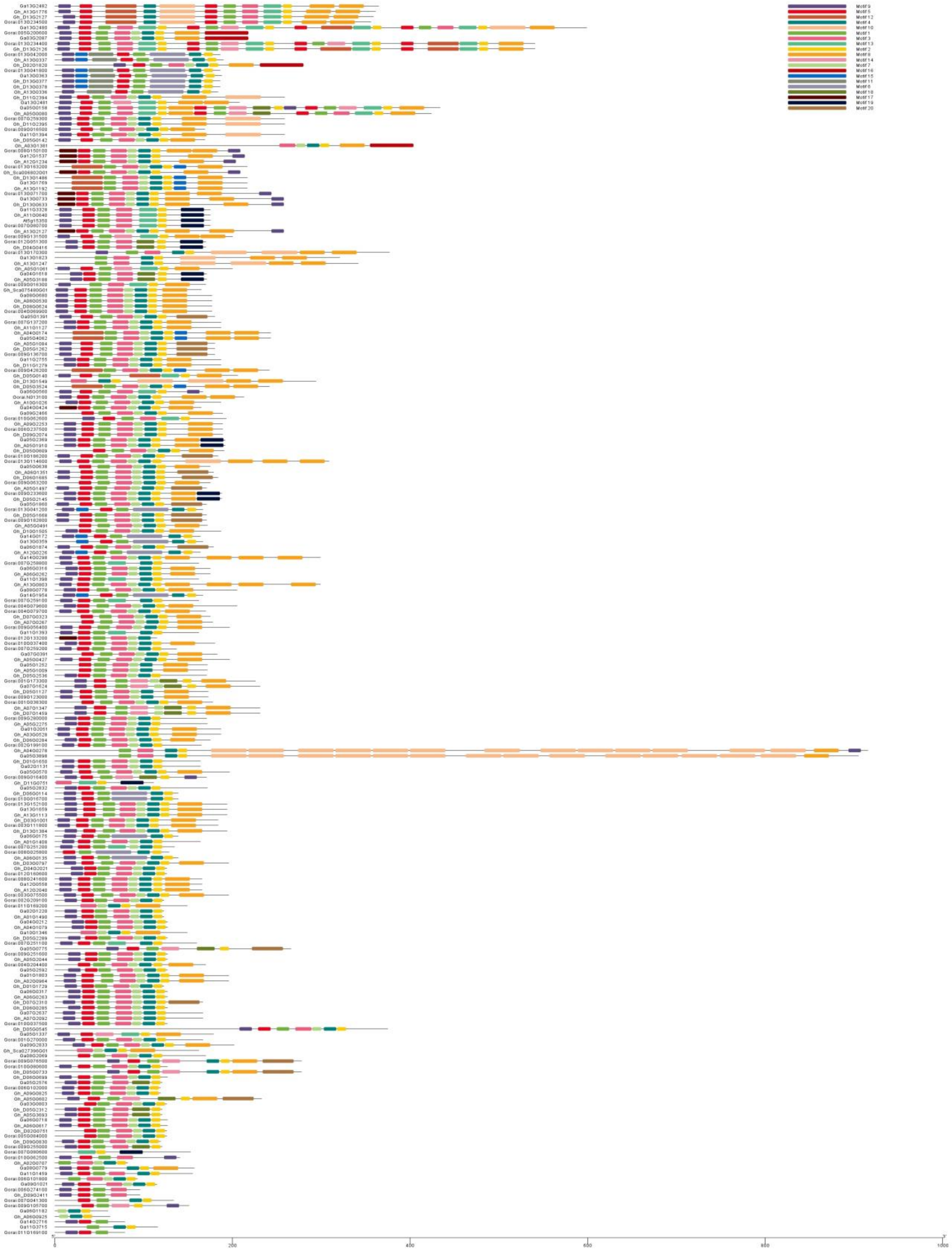

Figure S2: The conserved protein motifs of the amino acid sequences of PCs were analyzed by the MEME.
