# Supplementary figures and images for: Identification and Characterization of Phytocyanin Family Genes in Cotton Genomes
Source: Genes (Basel). 2023 Feb 28;14(3):611. doi: 10.3390/genes14030611 (PMC10048054; doi:10.3390/genes14030611)

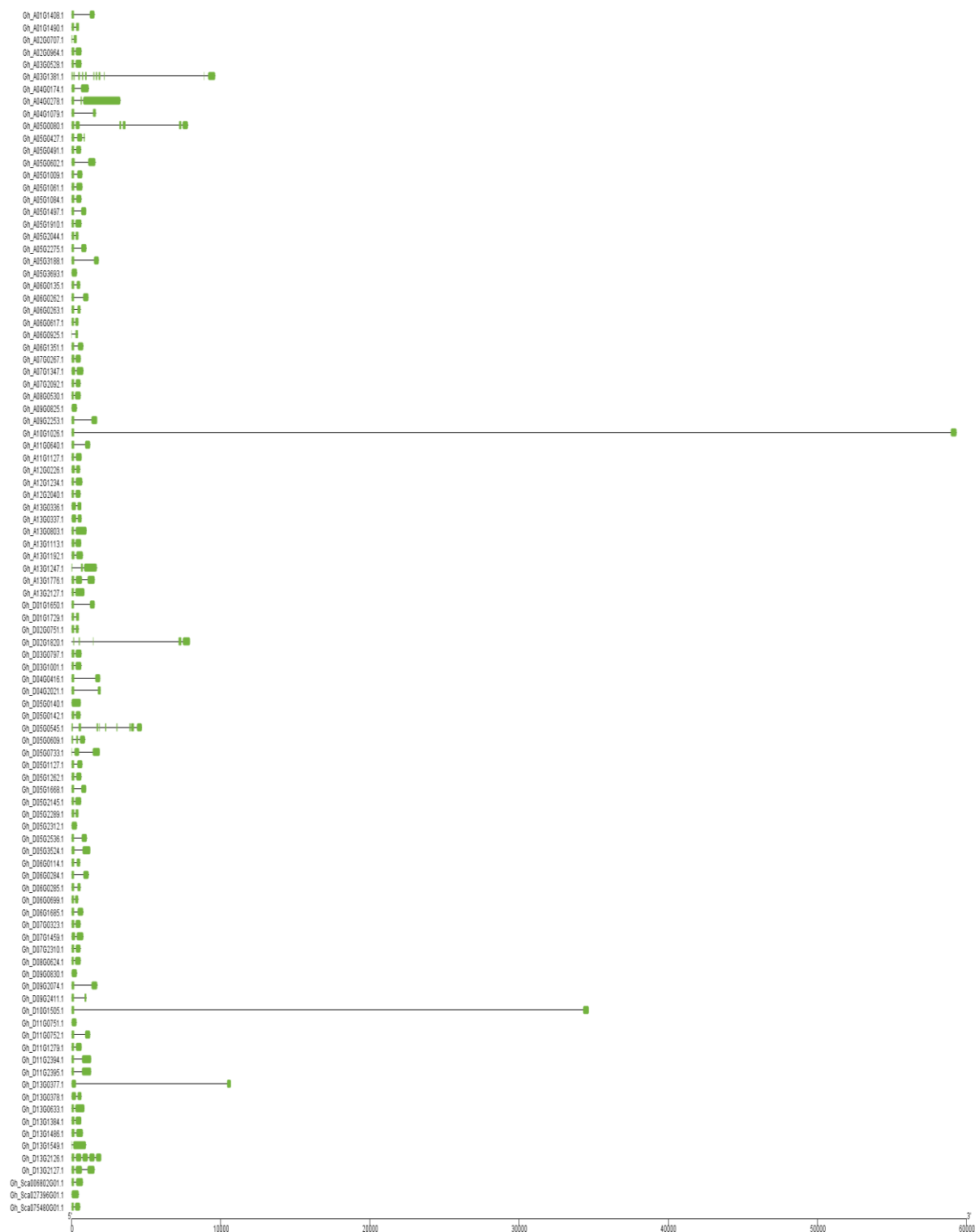

Figure S1: The gene structures of PCs. Gene structure analysis was performed by TBtools software.

Supplement: Supplementary file 1 [file genes-14-00611-s001.zip › Figure S1.pdf]

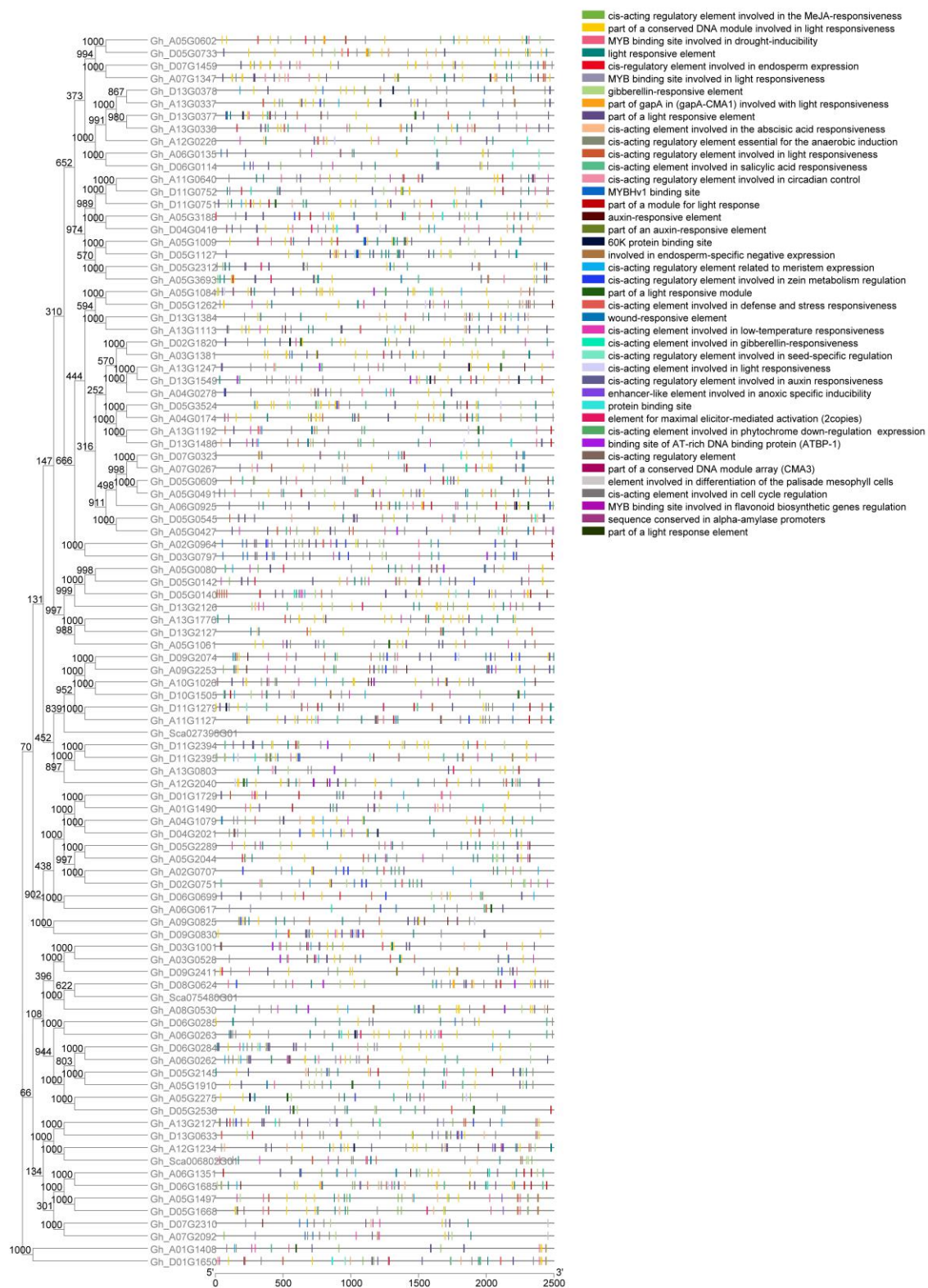

Supplement: Supplementary file 1 [file genes-14-00611-s001.zip › Figure S3.pdf]
